# *ATP1A3* mutations can cause progressive auditory neuropathy: a new gene of auditory synaptopathy

**DOI:** 10.1038/s41598-017-16676-9

**Published:** 2017-11-28

**Authors:** Kyu-Hee Han, Doo-Yi Oh, Seungmin Lee, Chung Lee, Jin Hee Han, Min Young Kim, Hye-Rim Park, Moo Kyun Park, Nayoung K. D. Kim, Jaekwang Lee, Eunyoung Yi, Jong-Min Kim, Jeong-Whun Kim, Jong-Hee Chae, Seung Ha Oh, Woong-Yang Park, Byung Yoon Choi

**Affiliations:** 1 Department of Otorhinolaryngology, National Medical Center, Seoul, Korea; 2 Department of Otorhinolaryngology, Seoul National University Bundang Hospital, Seongnam, Korea; 3 Samsung Genome Institute, Samsung Medical Center, Seoul, Korea; 4 Department of Health Sciences and Technology, Samsung Advanced Institute for Health Sciences and Technology, Sungkyunkwan University, Seoul, Korea; 5 Department of Otorhinolaryngology, Seoul National University Hospital, Seoul, Korea; 6 Division of Functional Food Research, Korea Food Research Institute (KFRI), Seongnam, Korea; 7 College of Pharmacy and Natural Medicine Research Institute, Mokpo National University, Muan, Korea; 8 Department of Neurology, Seoul National University Bundang Hospital, Seongnam, Korea; 9 Department of Pediatrics, Pediatric Clinical Neuroscience Center, Seoul National University Children’s Hospital, Seoul, Korea; 10 Department of Molecular Cell Biology, School of Medicine, Sungkyunkwan University, Seoul, Korea

## Abstract

The etiologies and prevalence of sporadic, postlingual-onset, progressive auditory neuropathy spectrum disorder (ANSD) have rarely been documented. Thus, we aimed to evaluate the prevalence and molecular etiologies of these cases. Three out of 106 sporadic progressive hearing losses turned out to manifest ANSD. Through whole exome sequencing and subsequent bioinformatics analysis, two out of the three were found to share a *de novo* variant, p.E818K of *ATP1A3*, which had been reported to cause exclusively CAPOS (cerebellar ataxia, areflexia, pes cavus, optic atrophy, and sensorineural hearing loss) syndrome. However, hearing loss induced by CAPOS has never been characterized to date. Interestingly, the first proband did not manifest any features of CAPOS, except subclinical areflexia; however, the phenotypes of second proband was compatible with that of CAPOS, making this the first reported CAPOS allele in Koreans. This ANSD phenotype was compatible with known expression of *ATP1A3* mainly in the synapse between afferent nerve and inner hair cells. Based on this, cochlear implantation (CI) was performed in the first proband, leading to remarkable benefits. Collectively, the *de novo ATP1A3* variant can cause postlingual-onset auditory synaptopathy, making this gene a significant contributor to sporadic progressive ANSD and a biomarker ensuring favorable short-term CI outcomes.

## Introduction

Auditory neuropathy spectrum disorder (ANSD) is a type of hearing loss characterized by electrophysiological findings of an impaired or absent response in auditory brainstem responses (ABR), despite evidence of intact outer hair cell function as supported by the presence of cochlear microphonics and/or detectable otoacoustic emission (OAE). Subjects with ANSD have varying degrees of hearing losses; however, they generally present poor speech recognition that is disproportionate to the degree of hearing loss and difficulty hearing in noise^1–3^. The etiologies of ANSD are highly diverse, including hypoxia, infection, kernicterus, cytotoxic oncologic drug, and genetic factors^4,5^.

As for prelingual genetic ANSD, there seems to be less diversity compared with postlingual-onset genetic ANSD. Only a few genes have been associated with prelingual genetic ANSD, including autosomal recessive *OTOF* (DFNB9; the otoferlin gene, NM_001287489) and *PJVK* genes (DFNB59; the pejvakin gene, NM_001042702)^6–9^. *GJB2* and mitochondrial 12SrRNA mutations have also been reported to be related to this phenotype^10–13^. Among them, *OTOF* mutations occupy a major part of prelingual ANSD in many Caucasian populations^14^. The predominance of *OTOF* mutations has also been established in Koreans as they were reported to account for up to 85% of the Korean population with prelingual ANSD with normal cochlear nerve^15^. As for postlingual-onset ANSD, a lot of syndromic forms that cause sensory and motor neuropathy have been documented in adults with ANSD, including Charcot-Marie-Tooth disease^2,16,17^, Friedreich’s ataxia^18,19^, deafness-dystonia-optic neuropathy (DDON) syndrome^20^, autosomal dominant optic atrophy (ADOA)^21,22^, and AUNX1 due to mutations in apoptosis-inducing factor^23,24^. However, there are not many reported genes related to non-syndromic, progressive ANSD with postlingual onset. Only mutations from *DIAPH3* as the cause of AUNA1^25,26^ have been anecdotally reported from familial ANSD cases, leaving a substantial portion of non-syndromic and sporadic forms of ANSD still unanswered with respect to the molecular etiology. *SLC17A8* encoding VGLUT3, if altered, has been shown to cause ANSD in mice^27,28^; however, to date, phenotypes of ANSD have not been reported in human subjects with *SLC17A8*-induced hearing loss^29,30^.

Herein, we aimed to explore the molecular etiology of three unrelated subjects manifesting sporadic, progressive ANSD with postlingual onset. We identified that sensorineural hearing loss (SNHL) in CAPOS syndrome (OMIM #601388), comprising of cerebellar ataxia, areflexia, pes cavus, optic atrophy, and hearing loss^31–37^, can fit into the category of ANSD and manifest in a non-syndromic manner.

## Results

### Diagnosis of ANSD

**Figure 1:**
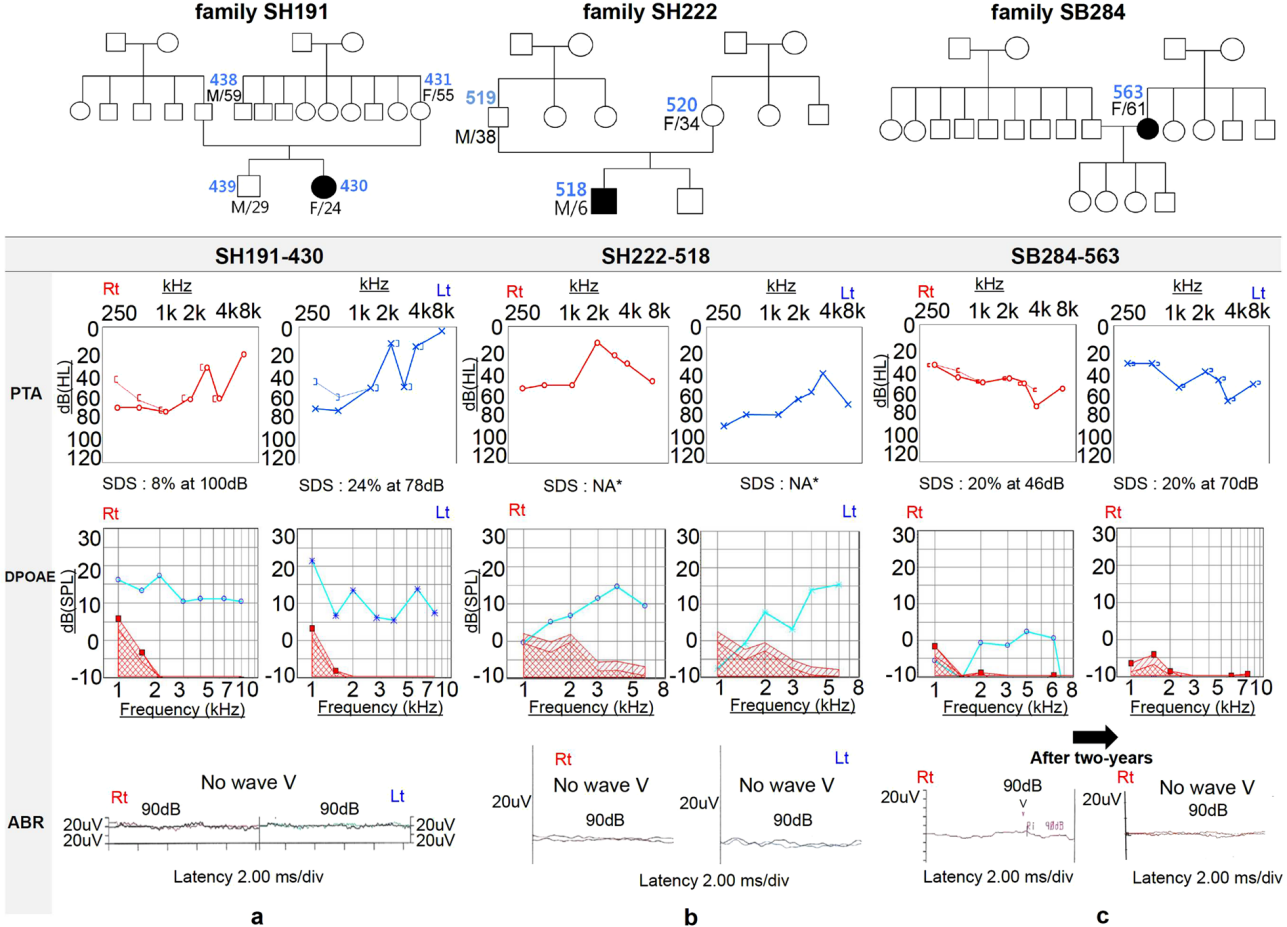
Pedigrees and audiological phenotype of the three probands. Pure tone audiograms of three ANSD subjects display moderate to severe sensorineural hearing loss, with poor speech discrimination score. DPOAE responses are observed, whereas ABR waveforms are absent or abnormal (**a**) SH191-430, (**b**) SH222-518, and (**c**) SB284-563. The patient SB284-563 shows detectable wave on ABR at 90 dB stimulus on the right ear and no response in both OAE and ABR after two-years. (PTA pure tone audiometry; SDS speech discrimination score; DPOAE distortion product optoacoustic emission; ABR auditory brainstem response; NA not available).

Among the 106 postlingually deafened subjects with moderate or higher degree of sporadic SNHL, at least three (3/106, 2.8%) unrelated subjects were documented to have ANSD (Fig. 1). The cochlear nerve in these subjects did not show any significant anatomic alterations.

### Molecular Genetic Testing and Delineation of Causative variant from the three subjects

We performed whole exome sequencing (WES) in three probands with sporadic ANSD and identified the most convincing causative variant using the bioinformatics filtering process that has previously been described^38^. This analytic step and the number of remaining candidates filtered at each step are listed (Table 1). Candidate variants were validated by standard Sanger sequencing and segregation study.

**Table 1:** Filtering process of whole exome sequencing data obtained from three auditory neuropathy spectrum disorder subjects.

Process	Detail	SH191-430	SH222-518	SB284-563
1	Raw VCF	537259	547209	525943
2	Annotated variants	261067	492276	525943
3	Exonic variants	12707	12839	24328
4	Rare variants(ExAC & KRGDB < 0.01)	973	1010	1021
5	Not reported or Flagged SNP	451	443	459
6	Inheritance pattern with high-quality variants and cut-off	165	120	186
	- *De novo* or dominant (MAF < 0.005)	137	101	160
	- Autosomal recessive- homozygote (MAF < 0.0005)	6	7	7
	- Autosomal recessive- compound heterozygote (MAF < 0.005)	22	12	19
7	Additional information(SIFT, PP2, GERP score, CLINVAR)	1*	1*	0

*c.2452 G > A: p.E818K of the *ATP1A3* gene (NM_152296.4, NP_689509.1, OMIM *182350).

**Figure 2:**
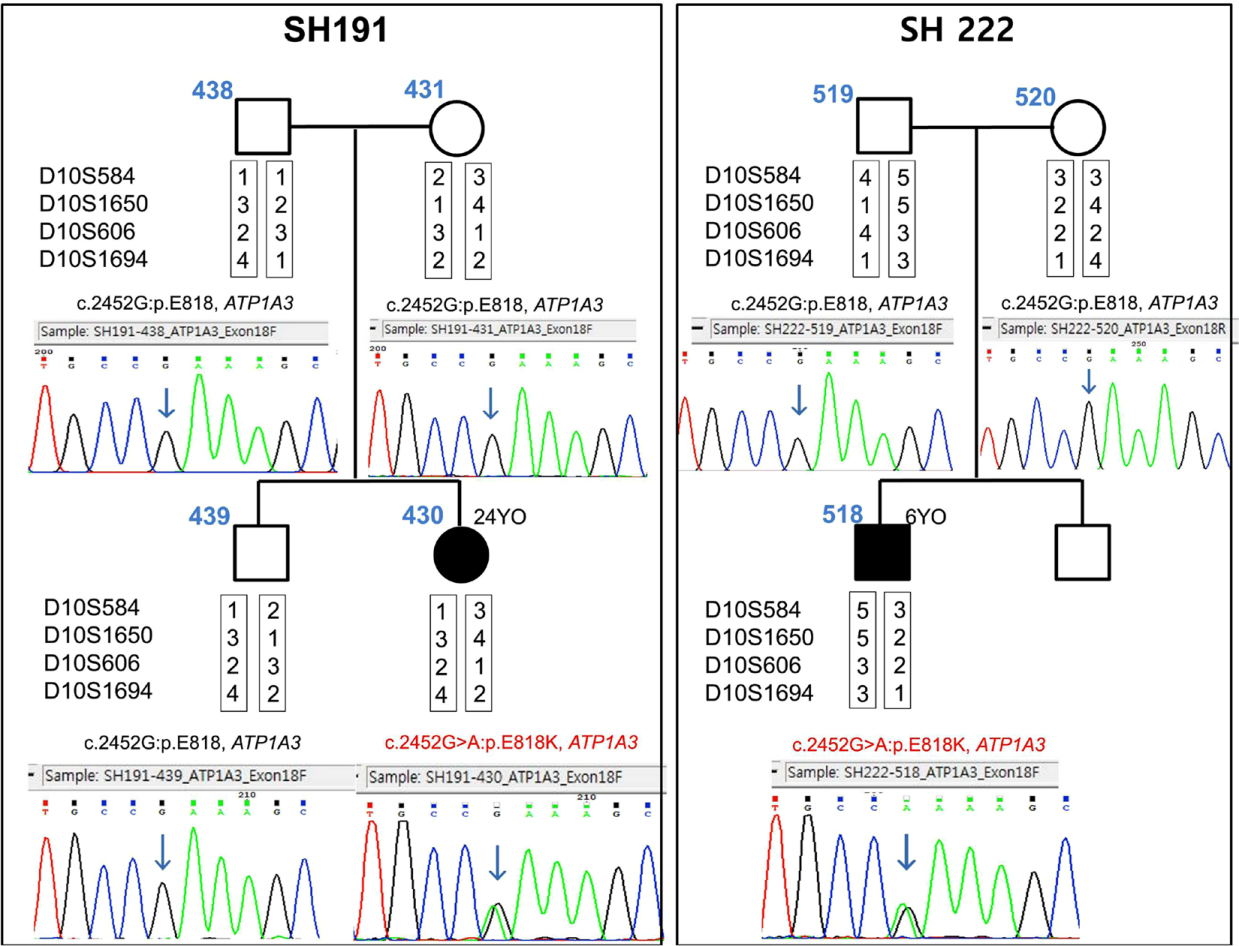
*De novo* occurrence of the causative variant. *De novo* occurrence of p.E818K of *ATP1A3* in families SH191 and SH222 is shown. Sanger sequencing traces of parents and probands are all provided. None of the parents (SH191-438, 431 and SH222-519, 520) from two families carry the variant residue, while the single heterozygous variant is noticed from both probands (SH191-430 and SH222-518). The results from reconstructed haplotypes derived from genotype results of four STR markers exclude non-paternity in families SH191 and SH222.

Interestingly, one heterozygous missense variant, c.2452 G > A: p.E818K of the *ATP1A3* gene (NM_152296.4, NP_689509.1, OMIM *182350), located on chromosome 19q13.2 and classified as ‘pathogenic’ according to CLINVAR (https://www.ncbi.nlm.nih.gov/clinvar/)^39^, was detected in two (SH191-430 and SH222-518) of the three ANSD subjects (Fig. 2). This variant was not detected from unaffected parents, indicating a *de novo* occurrence of an autosomal dominant variant, p.E818K, in both families (families SH191 and SH222) (Fig. 2). The paternity in these two families was confirmed by genotyping the four short tandem repeat (STR) markers, ensuring a *de novo* occurrence of p.E818K (Fig. 2). This p.E818K variant has previously been reported in a known CAPOS syndrome, comprising of cerebellar ataxia, areflexia, pes cavus, optic atrophy, and SNHL^27–30^. This variant has not been detected in the Exome Aggregation Consortium (ExAC; 121,412 alleles) and the Korean Reference Genome Database (KRGDB; 1,244 alleles). However, in the SB284 family, no convincing variants were found.

### Detailed medical history and clinical phenotype of three ANSD subjects

The first proband (SH191-430) was a 24-year-old woman that manifested SNHL when she was a teenager. Her SNHL was compatible with typical ANSD, as shown in Fig. 1a. Her pure tone audiogram (PTA) showed a reverse sloping configuration. Temporal bone computed tomography (TBCT) and internal auditory canal magnetic resonance imaging (MRI) confirmed that she had an intact cochlear nerve without any other anatomical abnormalities. She–along with her mother–denied any neurologic episodes, such as dystonia, ataxia, and visual disturbance. Detailed neurologic examination by an experienced neurologist confirmed the absence of co-morbid neuropathy, except for nearly absent deep tendon reflex (DTR). A detailed fundus examination and visual acuity test of SH191-430 did not reveal any abnormality, and SH191-430 refused to take visual evoked potentials (VEP). Until further examination, her SNHL had been classified as non-syndromic ANSD.

The second proband (SH222-518) was a 6-year-old boy when he first visited the ENT clinic. He successfully passed the newborn hearing screening test, which was performed with automated ABR, suggesting that his SNHL was not congenital. His development had not been noteworthy, except his experience of two acute episodes of neurologic symptoms and signs, including lethargy, instability while sitting, ataxia, dysarthria, visual disturbance with dancing nystagmus, and hearing loss after febrile illness at the age of 3 and 5 years. His SNHL was entirely compatible with ANSD, considering a detectable OAE response without ABR response (Fig. 1b). At that time, the findings from cerebrospinal fluid tapping and MRI were normal. He recovered steadily from all symptoms with minimal neurologic sequelae, mild temporal pallor in color fundus photography (Supplementary Fig. S1), and normal response in VEP. However, his SNHL deteriorated over time.

The third proband (SB284-563) was a 61-year-old female. Her SNHL started in her early thirties. Like the first proband, she did not have any neurologic abnormalities, except hearing loss. She had moderate hearing loss on PTA, 45 dB on the right and 40 dB on the left, with a disproportionately low word recognition score of 20%, bilaterally. In line with this finding, she showed a detectable ABR wave only at 90 dB stimulus on the right ear, despite the presence of a positive OAE response back in 2014, which indicated that her SNHL was compatible with ANSD. Two years later, both her OAE and ABR responses disappeared (Fig. 1c).

The neurologic features of these three ANSD subjects are presented in Table 2.

**Table 2:** Overview neurologic features of three probands related with CAPOS syndrome.

Subject	SH191-430	SH222-518	SB284-563
Gender	F	M	F
Current age	24	6	61
Onset of hearing difficulty	10’s	3 years	30’s
CAPOS related feature at acute episode			
- Age at episodes	−	3 and 5 years	−
- Trigger	−	fever	−
- Ataxia	−	+	−
- Abnormal eye movement	−	+ (opsoclonus)	−
- Dysarthria	−	+	−
- Dysmetria	−	+	−
- Dysphagia	−	−	−
- Visual disturbance	−	+	−
- Tremor	−	−	−
- Seizure	−	−	−
Findings at latest examination			
- Gait	normal	slightly ataxic	normal
- Areflexia	+	+	−
- SSEP (somatosensory evoked potential)	normal	(not available)	normal
- NCS (nerve conduction velocity)	normal	normal	normal
- Pes cavus	−	−	−
- Optic atrophy	−	+	−

### Short term follow-up results after cochlear implantation

**Figure 3:**
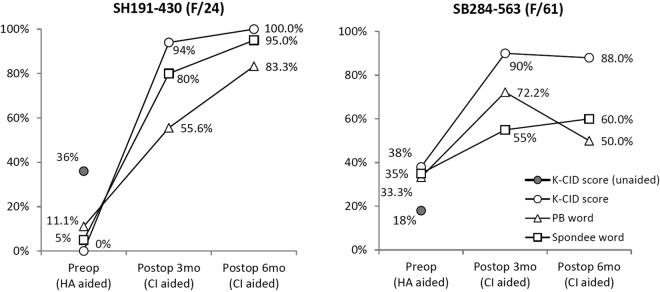
Pre- and postoperative cochlear implant speech performance. Both cochlear implantees show markedly increased performance on speech evaluation with K-CID as well as on PB and spondee word scores during the first three postoperative months. SH191-430 continues to display improvement in speech performance over the next three months, while improvement of SB284-563 slows down during the period. (K-CID Korean version of the Central Institute of Deafness; PB word phonetically balanced word; HA hearing aid; CI cochlear implant).

Two adult ANSD subjects (SH191-430 and SB 284-563) underwent cochlear implantation (CI) since they were unable to benefit from hearing aids. The first subject (SH191-430) had the highest difficulty in speech discrimination with hearing aid, especially with background noise. She scored worse on the Korean version of the Central Institute of Deafness (K-CID) test with hearing aid, which decreased from 36% without hearing aid to 0% with hearing aid. Both subjects had CI on the right ear after getting replicable, albeit not perfect, waveforms on trans-tympanic electrically evoked ABR (EABR) (Supplementary Fig. S2). Both subjects showed striking progress in speech recognition, scoring 90–94% on K-CID without visual cue at 3 months postoperatively (Fig. 3). Evaluation of the speech performance at 6 months postoperatively displayed continuous improvement in this subject (SH191-430) carrying p.E818K of *ATP1A3* (Fig. 3).

## Discussion

The etiologies of ANSD are highly diverse; approximately 40% of cases have an underlying genetic basis^5^, whereas relatively a small number of variants has been evaluated to cause postlingual ANSD. We, for the first time, discovered that a *de novo* variant of *ATP1A3* could be a prevalent and important etiology of sporadic ANSD with postlingual onset.

The *ATP1A3* gene has been reported mainly in association with alternating hemiplegia of childhood (AHC, AHC2, OMIM #614820) and rapid-onset dystonia Parkinsonism (RDP, DYT12, OMIM #128235)^35,40–42^. Among various *ATP1A3* mutations, two variants, p.D810N and p.E815K, have been found recurrently in more than 50% of patients with AHC, while no distinct mutations have been found in patients with RDP^35^. A different variant of this gene, p.E818K (c.2452 G > A), which has not been detected in the context of two main diseases associated with *ATP1A3*, has been reported to cause the third allelic disorder, CAPOS syndrome without exception in several reports^31–37^. CAPOS syndrome was first described in 1996 by Nicolaides *et al*. in a British family^43^. To date, this single variant has been identified exclusively from all nine unrelated CAPOS families, as described previously with 100% frequency. Unlike the previous two diseases, SNHL with sudden onset and progressive nature is a distinctive disabling feature of CAPOS syndrome. However, there has not been a rigorous study on the type of SNHL thus far.

The second subject, SH222-518, had passed the newborn hearing screening test by automated ABR; however, his hearing impairment was initiated and became prominent with a definite progression through two febrile events. Together with accompanying symptoms and signs, such as ataxia and visual disturbance, the detection of p.E818K in *ATP1A3* led to the diagnosis of CAPOS syndrome. Different variants of this gene have been reported to cause RDP and AHC, without the companion of hearing or visual disturbance^31,35^. On the other hand, the first subject, SH191-430, who shared the same p.E818K variant, denied any neurological symptoms, except areflexia that was elucidated only after a rigorous neurological examination by an expert neurologist. This subject would have been classified as non-syndromic without a comprehensive neurologic examination by an expert. This is not surprising considering that there have been some reports showing subjects with CAPOS syndrome exhibiting mild symptoms resulting in recovery with minimal residual ataxia during walking and standing on one foot^31^. This implicates that extensive history taking and rigorous neurologic examination would be an essential part in the diagnosis of postlingual and progressive ANSD.

Our present study reports the tenth CAPOS family (SH222) and the 27^th^ CAPOS patient (SH222-518) in the literature. This is the first report of a CAPOS allele in Koreans. High mutability of this residue, as shown by frequent *de novo* occurrence of p.E818K (c.2452 G > A) of *ATP1A3* both in Caucasians^31^ and in Koreans, indicates that the effect of this mutational hotspot is not limited to certain populations but would rather be global. This is noteworthy because other allelic disorder of *ATP1A3*, RDP, has also been reported to be frequently caused by *de novo* variants^44^. Indeed, with the advent of next generation sequencing (NGS) technologies, recent studies using NGS have elucidated the important role of *de novo* variants in several important neurological disorders^45–47^. To date, it is unclear exactly why *de novo* variants frequently arise in *ATP1A3*. Recently, the rate of *de novo* mutations has been reported to be heavily influenced by the age of the father^48^; however, that study failed to account for all *de novo* cases. There might be a genetic context in *ATP1A3* favoring the occurrence of *de novo* variants, awaiting further clarification. Our present study also unveils that a phenotypic spectrum related to p.E818K of *ATP1A3* can be extended to include nearly non-syndromic postlingual ANSD, not limited to the canonical CAPOS syndrome.

*ATP1A3* encodes the α3 catalytic subunit of Na^+^/K^+^ ATPase (NKA), which is a membrane-bound transporter using ATP to transport three Na^+^ outwards in exchange for two K^+^ into the cell, resulting in a negative transmembrane potential. Regarding the contribution of NKA on hearing, it has mainly been studied in relation to endocochlear potentials (EP). The inhibitors of NKA, such as ouabain, are known to reduce EP^49,50^. There are four α isoforms identified in vertebrates^51,52^. NKAα1 has been identified in the strial marginal cell, and NKAα2 in the lateral wall fibrocyte of the stria vascularis, which is the source of EP^50,53^. Indeed, heterozygous deletion of NKAα1 or NKAα2 in mice resulted in the reduction of EP and progressive, age-dependent hearing loss^54^. Accordingly, the *ATP1A2* gene (encodes NAKα2), which has previously been reported to be the genetic cause of familial hemiplegic migraine type 2 (FHN2, OMIM #602481), has recently been reported to be associated with progressive hearing loss with migraine^55^. Specifically, co-segregation of a novel variant, c.571 G > A, in the *ATP1A2* gene with migraine and hearing loss in a family was shown, although the pathogenic potential of the c.571 G > A variant was not confirmed. Contrastingly, the α3 isoform is rather neuron-specific, albeit being co-expressed with ubiquitous α1 in most neurons^56–58^. NKAα3 is known to play an important role in the rapid restoration of increased intracellular Na^+^ concentration after high neuronal activity in the nervous system^56,59^. Therefore, disrupted NKAα3 activity leads to a failure in regaining the resting membrane potential after excitatory activity, and it has been proposed to cause paroxysmal neurologic events observed in two main *ATP1A3*-related syndromes^34,35,60^. Several experimental studies evaluated the pump activity as well as the effect of pathologic variants of *ATP1A3*. They showed that these mutations caused a reduction in catalytic activity and a failure to generate pump current. This finding is consistent with the inability to bind to K^+^ ions and a reduction in the Na^+^ affinity for activating phosphorylation^61^.

Nonetheless, the exact role of NKAα3 in hearing and how mutations in *ATP1A3* cause hearing loss have not been fully elucidated to date. In a study focusing on the NKAα expression pattern in Organ of Corti and the neuronal element of cochlea, it has been demonstrated that NKAα3 is expressed abundantly in the membranes of type I afferent terminals that are in contact with the inner hair cells, spiral ganglion somata, and medial efferent terminals contacting the outer hair cells^50^. NKAα1 has been identified in the supporting cells neighboring the inner hair cells, and NKAα2 has not been detected in either the organ of Corti or spiral ganglion^50^. This expression pattern of NKAα3 in the cochlea has been shown to be compatible with an observation in which perfusion of ouabain, a specific inhibitor of NKAα3 in the cochlea, directly administered through the round window in gerbil, resulted in a reduced or completely abolished compound action potential but did not reduce EP^50,62,63^. This suggests that NKAα3 may have a more direct role in the regulation of signal transmission in the synapses with hair cells and spiral ganglion cells, rather than having a contribution to generate EP. Therefore, it is possible for the alteration of NKAα3 to lead to the clinical and laboratory features compatible with ANSD.

ANSD is a heterogeneous disorder that may result from various etiologies and different lesion sites, ranging from the inner hair cells and synapses to auditory nerve and central cortex. Inherent from such pathologic characteristics of ANSD, the behavioral threshold measures are not consistent with other measures of auditory function, such as ABR and speech understanding scores. Furthermore, it poses a challenge when choosing an appropriate method of auditory rehabilitation and predicting the outcome^4,64,65^. Due to the uncertainties of outcomes after CI, it is important to select patients who are expected to have good results from CI. EABR is widely used to evaluate the integrity of auditory pathway and predict the outcome of CI^66–68^. Among the various subtypes of ANSD classified by their predominant lesion, auditory synaptopathy has been reported to have excellent CI results, and EABR is typically present in this subtype^3,21,65,69–71^. However, the interpretation of waveforms is somewhat subjective, and preoperative trans-tympanic EABR is not consistently reliable for predicting the outcome of CI^72^. It is not unprecedented to benefit significantly from CI even in cases without any detectable responses from both preoperative trans-tympanic and postoperative intracochlear EABR tests^73,74^. Therefore, genetic diagnosis that enables precise localization of the pathologic site in the auditory pathway has been recognized to be very important.

Mutations in the *OTOF* gene, a causative gene of auditory synaptopathy, may cause a dysfunction in Ca^2+^-triggered exocytosis of a neurotransmitter in the ribbon synapses of the inner hair cells^75–77^. Therefore, a direct electrical stimulation of the spiral ganglion cells with CI bypassing the main lesion can restore well-synchronized neural responses that propagate through the intact auditory nerve. Another ANSD with good results on CI is a syndromic ANSD accompanied by autosomal dominant optic atrophy (ADOA, OMIM #165500) due to *OPA1* variations^11,21,64^. Although the specific function of the *OPA1* gene regarding hearing has not been fully elucidated to date, detailed assessment with trans-tympanic electrocochleography and electrically-evoked response following CI indicated that the site of the lesion is at a terminal portion of auditory nerve fibers with the potential to progress proximally at advanced stages^11^. *OPA1* mRNA and protein were found in the hair cells as well as in the neuronal fibers innervating the inner hair cells, ganglion cells of the cochlea, and vestibular organ^78^. This is significantly overlapped with the expression site of the *ATP1A3* protein, NKAα3. Although the function of NKAα3 has not been fully revealed, we can postulate from the expression pattern of this protein that the main pathologic lesion site would be a synapse between the inner hair cells and the afferent nerve fibers, or at most spiral ganglion neurons. If the lesion is mainly the former, then we can expect good CI results, similar to the ANSD associated with *OTOF* and *OPA1*. In accordance with this expectation, one (SH191-430) of our two ANSD subjects carrying p.E818K (c.2452 G > A) of *ATP1A3* underwent CI and showed excellent performance at 3 and 6 months after CI, although longer observation is needed (Fig. 3). The third subject (SB284-563), whose causative variants were not identified, also underwent CI and showed successful outcome. Therefore, it can be deduced that the main pathology of ANSD in this subject may be limited to either pre-synaptic area or synapsis itself, rather than the nerve itself or a more central lesion.

What merits further attention is that the *ATP1A3* gene is expressed not only in the synapse, but also in the spiral ganglion. Although the spiral ganglion neuron is electrically simulated by CI, possible progressive degeneration of the spiral ganglion neuron could still occur due to a disruption in the function of *ATP1A3*. In such a case, the best functional outcome of CI observed at the short-term follow-up may decrease over time. Therefore, a continuous follow-up of CI over a longer period is mandatory. Conversely, earlier stimulation of the spiral ganglion cell neuron by CI may retard or attenuate the degeneration of the neuron. This perspective has already been applied for the interpretation of discrete CI outcomes related to mutations of *TMPRSS3*, which is a representative gene expressed in the spiral ganglion neuron^79–81^. The outcomes of CI performed earlier in life due to rapid loss of residual hearing caused by more pathologic *TMPRSS3* mutations was favorable, while the result from late implantees carrying *TMPRSS3* mutations was not as favorable as the expectation^81^. Given the similarity of the expression site between *ATP1A3* and *TMPRSS3*, a long-term follow-up of CI performance is warranted to better determine if the current benefit from CI decreases over time.

The exact frequency of post-lingual onset ANSD is still unknown. The prevalence of ANSD has been reported mainly in pediatric groups, ranging from 2% to 15% of children with permanent SNHL^82–86^. Although the prevalence of ANSD among the post-lingual SNHL has not been reported separately, the proportion of adult patients among the entire ASND population has been mentioned in some studies. Sininger and Oba reported that 25% of ANSD patients experienced their first symptoms after the age of ten^1^; other studies by Starr *et al*. and Berlin *et al*. showed that in 12~20% of cases, onset was experienced in adulthood, most of whom had multiple neuropathies due to Charcot-Marie-Tooth disease^2,87^. In our present study, three patients (2.8%) out of 106 sporadic cases of postlingual moderate and more degree of SNHL were diagnosed with ANSD. This prevalence is markedly lower when compared with the pediatric group. It is likely that many post-lingual patients with hearing difficulty did not undergo enough audiological and electrophysiological tests to fully confirm ANSD. Moreover, this may be even further underestimated because OAE response, which was once considered a prerequisite, might have been absent or disappeared over time^2,88–90^. In our study, the disappearance of OAE response from the third subject, SB284–563, over a period of 2 years (Fig. 1) supports this speculation.

In our present study, the single *de novo* variant, p.E818K of *ATP1A3*, accounted for two of the three (2/3, 67%) postlingual-onset ANSD, suggesting that this specific variant of *ATP1A3* could be a significant contributor to postlingual-onset ANSD, not only in Koreans but also in other populations. Moreover, an evaluation of postlingual-onset ANSD subjects should require a comprehensive neurologic examination and meticulous history taking to ensure that no episodes of ataxia and visual disturbance triggered by a fever go unnoticed. Molecular genetic testing of this particular variant, p.E818K of *ATP1A3*, can be helpful to delineate ANSD cases which are related to subtle cases of CAPOS syndrome.

## Conclusions

Here, we showed that recurring *de novo* mutation of *ATP1A3* can cause progressive ANSD with postlingual onset and with either absence or presence of syndromic features. This confirms that *ATP1A3* is an important and prevalent causative gene for progressive ANSD with postlingual onset. The presence of this variant can potentially serve as not only an etiologic biomarker, but also as a prognostic biomarker that allows for the identification of a subset of postlingual-onset ANSD subjects who can significantly benefit from CI, at least during a short-term follow-up period.

## Material and Methods

### Ethical considerations and subject evaluation

This study was approved by the institutional review boards of Seoul National University Hospital (IRBY-H-0905-041-281) and Seoul National University Bundang Hospital (IRB-B-1007-105-402). Informed consent was obtained from all participants in this study. Written informed consent for child participants was obtained from their parents or guardians. All methods in this study were performed in accordance with the relevant guidelines and regulations.

Medical and developmental histories were collected for 106 sporadic patients recruited between 2010 and 2017, who showed postlingual-onset, moderate or higher degree of SNHL. Syndromic cases were excluded. The audiometric evaluation with PTA, OAE, and ABR were carried out for the clinical diagnosis of ANSD. Internal auditory canal MRI and TBCT were also performed to identify anatomically intact cochlear nerve and any other anatomical abnormalities related to hearing loss. Comprehensive neurologic evaluation was performed by a neurology specialist to see if there is any accompanied neuropathy. The diagnostic criteria for ANSD as described by Rapin *et al*. were applied; (1) understanding of speech worse than predicted from the degree of hearing loss on their behavioral audiograms; (2) recordable otoacoustic emissions and/or cochlear microphonic; and (3) absent or atypical auditory brain stem responses^91^.

### Molecular genetic analysis

#### DNA preparation and whole-exome sequencing

Whole blood (10 ml) was obtained from two probands and, if possible, their siblings and parents for the segregation study. Genomic DNA samples were extracted from the peripheral blood via standard procedures, as previously described^92^, and were subjected to WES. A SureSelect 50 Mb Hybridization and Capture kit was used for WES, and sequencing was performed using a HiSeq2000. The read length for the paired-end reads was 100 bp.

#### Detection of single-nucleotide variants and insertion/deletion polymorphisms

The reads were aligned with the human genome reference sequence (hg19) as proposed by Burrows-Wheeler Aligner, version 0.7.5^93^ with the “MEM” algorithm. We used SAMTOOLS version 1.2 for sorting and indexing the SAM/BAM files^94^. Picard-tools version 1.127 (http://broadinstitute.github.io/picard) was used to mark duplicates. We used the IndelRealigner and BaseRecalibrator from Genome Analysis Toolkit (GATK) version 3.1-3^95^ based on known single-nucleotide polymorphisms (SNPs) and insertions/deletions (indels) from the Database of Single Nucleotide Polymorphisms (dbSNP, build138), Mills, and 1000 G gold standard indel b37 sites and 1000 G phase I indel b37 sites for realigning the reads and base recalibration. The targeted gene variants were called using the Unified Genotyper in GATK and also were recalibrated by GATK based on dbSNP138, Mills indels, HapMap, and Omni and filtered at 99.0 truth sensitivity level. Finally, we used ANNOVAR to annotate the variants^96^.

#### Filtering process to identify candidate variants

We went through the filtering process as previously described^38^. In brief, we selected the variants in the coding region and with allele frequencies of <0.01, which were reported in the Exome Aggregation Consortium (ExAC; 121,412 alleles; http://exac.broadinstitute.org) and the Korean Reference Genome database (KRGDB; 1,244 alleles; http://152.99.75.168/KRGDB). Then, we filtered out the variants reported in dbSNP138, but included flagged SNPs. We chose the variants that were matched with one of the three possible inheritance patterns that could be possible in the sporadic form of genetic hearing loss. They were filtered to discard any variants with false-positive results based on minor allele frequency (MAF) cut-off ^97^ and with variant allele frequency (VAF) of heterozygosity of less than 0.3; however, the criteria were selectively adjusted to be less than 0.1 when a variant was located in a homologous locus or a gene with a pseudogene or homologous between family members. Finally, the scores of SIFT (http://sift.jcvi.org/), PolyPhen2 (http://genetics.bwh.harvard.edu/pph2/) and GERP (http://mendel.stanford.edu/SidowLab/downloads/gerp/) and CLINVAR (https://www.ncbi.nlm.nih.gov/clinvar/)^39^ were used to assess the pathogenicity of candidates.

### Confirmation of paternity

To exclude non-paternity, genotypes of four unlinked STR markers (D10S584, D10S1650, D10S606, and D10S1694) from family members were analyzed, using a previously described method^98^. The four STR markers were chosen because they were shown in a previous study to be hypervariable and informative among Koreans^98^. A definite haplotype was reconstructed based on the information from parental sample and siblings.

### Data availability

All data, whether generated or analyzed during this study, were included in this published article (and its Supplementary Information files).

## Electronic supplementary material

Supplementary Figure S1 and S2
