# Supplementary material for: ATP1A3 mutations can cause progressive auditory neuropathy: a new gene of auditory synaptopathy
Source: Sci Rep. 2017 Nov 28;7:16504. doi: 10.1038/s41598-017-16676-9 (PMC5705773; doi:10.1038/s41598-017-16676-9)
Supplement: Supplementary file 1 — Supplementary Figure S1 and S2 [file 41598_2017_16676_MOESM1_ESM.pdf]

ATP1A3 mutations can cause progressive auditory neuropathy: a new gene of auditory synaptopathy

Kyu-Hee Han[1], Doo-Yi Oh[2], Seungmin Lee[2], Chung Lee[3,4], Jin Hee Han[2], Min Young Kim[2], Hye-Rim Park[2], Moo Kyun Park[5], Nayoung K.D. Kim[3], Jaekwang Lee[6], Eunyoung Yi[7], Jong-Min Kim[8], Jeong-Whun Kim[2], Jong-Hee Chae[9], Seung Ha Oh[5] ,Woong-Yang Park[3,4,10], Byung Yoon Choi[2,*]

[1]Department of Otorhinolaryngology, National Medical Center, Seoul, Korea

[2]Department of Otorhinolaryngology, Seoul National University Bundang Hospital, Seongnam, Korea

[3]Samsung Genome Institute, Samsung Medical Center, Seoul, Korea

[4]Department of Health Sciences and Technology, Samsung Advanced Institute for Health Sciences and Technology, Sungkyunkwan University, Seoul, Korea

[5]Department of Otorhinolaryngology, Seoul National University Hospital, Seoul, Korea

[6]Division of Functional Food Research, Korea Food Research Institute (KFRI), Seongnam, Korea

[7]College of Pharmacy and Natural Medicine Research Institute, Mokpo National University, Muan, Korea

[8]Department of Neurology, Seoul National University Bundang Hospital, Seongnam, Korea

[9]Pediatric Clinical Neuroscience Center, Department of Pediatrics, Seoul National University Children's Hospital, Seoul, Korea

[10]Department of Molecular Cell Biology, School of Medicine, Sungkyunkwan University, Seoul, Korea

***Correspondence to**: Byung Yoon, Choi, MD, PhD

Department of Otorhinolaryngology, Seoul National University Bundang Hospital, 82 Gumi-ro 173 beon-gil, Bundang-gu, Seongnam 463-707, Republic of Korea

Email: choiby@snubh.org, choiby2010@gmail.com

Tel: +82-31-787-7406

Fax: +82-31-787-4057

SUPPLEMENTARY INFORMATION

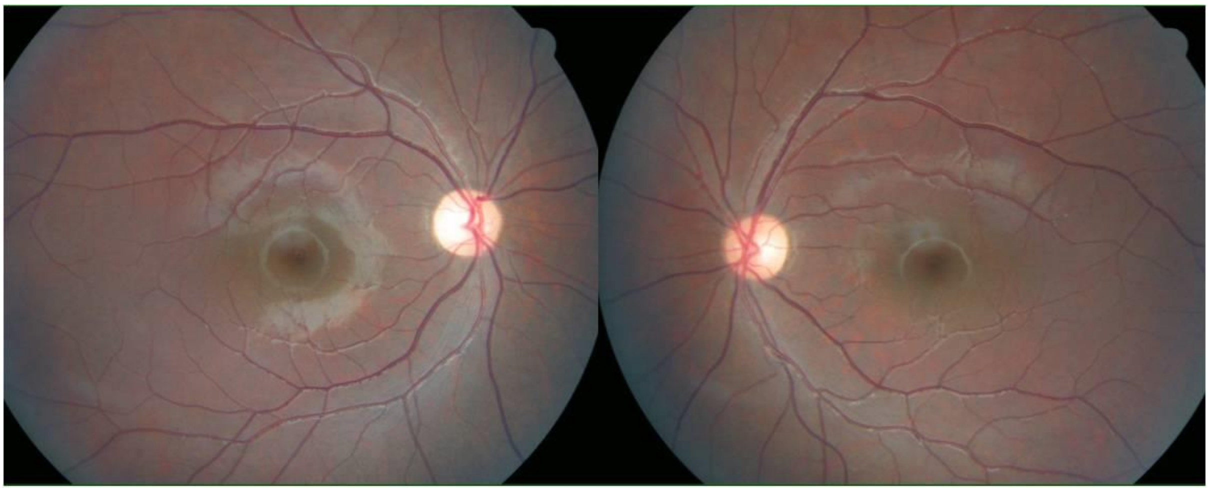

Supplementary Figure S1. Fundus images of patient SH222-518 The color fundus photographs of right eye (left) and left eye (right) taken at age of six revealed mild temporal pallor in both eyes.

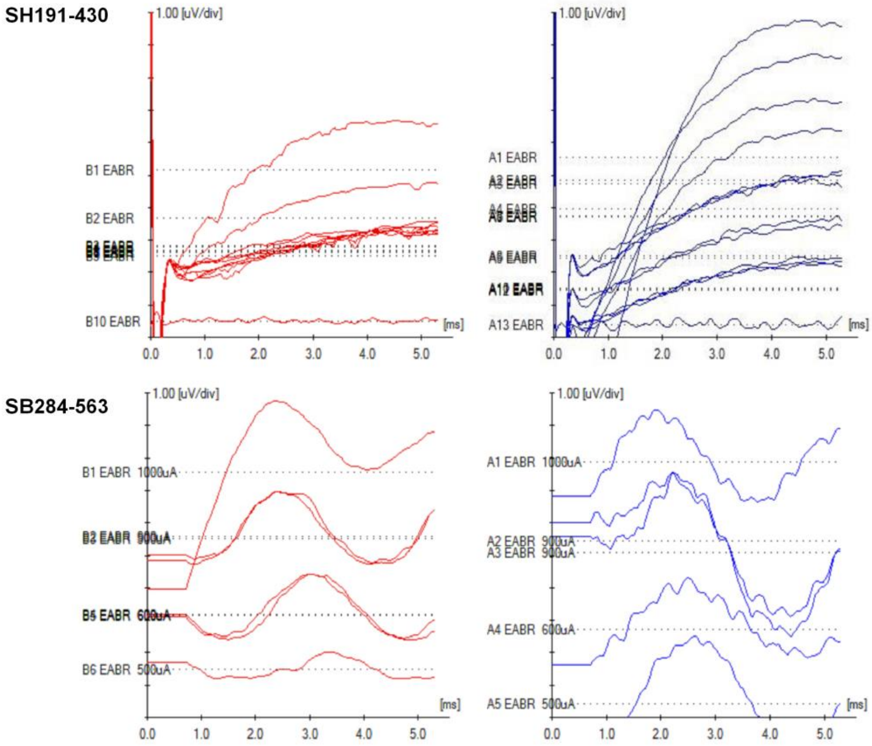

Supplementary Figure S2. Preoperatively measured electrically evoked auditory brainstem response Auditory brainstem response with trans-tympanic electrical stimulation presented reproducible waveforms bilaterally in two cochlear implantees (SH191-430 and SB 284-563).
